# Comparative assessment of the quality of commercial black and green tea using microbiology analyses

**DOI:** 10.1186/s12866-017-1142-z

**Published:** 2018-01-05

**Authors:** Federica Carraturo, Olga De Castro, Jacopo Troisi, Adriana De Luca, Armando Masucci, Paola Cennamo, Marco Trifuoggi, Francesco Aliberti, Marco Guida

**Affiliations:** 1 Department of Biology, University of Naples Federico II, Via Cinthia, 80126 Naples, Italy; 2 Department of Biology, University of Naples Federico II, Via Foria 223 – Orto Botanico, 80139 Naples, Italy; 3 Department of Medicine and Surgery, University of Salerno, Via Giovanni Paolo II 132, 84084 Salerno, Italy; 4 Department of Veterinary Medicine and Animal Production, University of Naples Federico II, Via Delpino 1, 80137 Naples, Italy; 5 S.G. Moscati Hospital, Contrada Amoretta, 83100 Avellino, Italy; 6 Faculty of Humanities, University of Suor Orsola Benincasa, Santa Caterina da Siena 37, 80135 Naples, Italy; 7 Department of Chemical Sciences, University of Naples Federico II, 80126 Naples, Italy

**Keywords:** Bacteria, Tea, *Camellia sinensis*, DNA barcoding, Fungi, Microbial contaminant, Ochratoxin A

## Abstract

**Background:**

Drinking tea constitutes a tradition which is deeply rooted in the culture of several countries. Moreover, in recent years, tea consumption is growing all over the world. Improper herbal tea storage (long periods, humid environments) represents a relevant health hazard for consumers because of the growth of bacteria and molds.

**Results:**

This study analyzed 32 samples of commercially available black and green teas – purchased from southern Italy markets and online-shops – and the monitoring of microbiological quality of the tea bag content was performed. Evaluations were conducted with the aim of characterizing pathogens indicated by the European and American guidelines (total bacterial count, fungi and *Escherichia coli*) and on the research of *Pseudomonas* spp. and *Clostridium perfringens*. The presence of ochratoxin A in tea matrix-leaves and infusions was further assessed, using a validated and accredited HPLC-FLD method. Microbial loads, for over 80% samples, ranged from 1.0 × 10^2^ to 2.8 × 10^5^ CFU/g tea: most of identified microorganisms were classified as *Bacillaceae*. The utilization of rapid detection and identification methods (PCR and sequencing), allowed the characterization of strains of *Pseudomonas psychrotolerans*, *Staphylococcus warneri*, *Pantoea gaviniae* and the isolation of one strain of *Clostridium perfringens*, whose ability to produce toxins can result in harmful outcomes for consumers. Fungi were isolated from 70% samples: the most prevalent molds were *Aspergillus niger* strains, followed by *Aspergillus tubingensis*. Ochratoxin A was detected in 22 of 32 tea solid samples investigated: concentrations resulted over the indicated limits for food products for 50% samples.

**Conclusions:**

Results obtained demonstrated the need to develop targeted regulations for the safety of herbal teas.

## Background

The most common tea types, basing on processing, are raw (usually identified as green tea) and ripened (or fermented, black) tea [1]. Fresh tea leaves undergo wilting and heat fixing processes, to sensibly reduce the water content and consent the development of the tea typical flavours. These procedures are carried out trying to avoid microorganisms spoilage due to humid environments and variable temperatures [2]. Despite the positive role of associated bacteria and molds in improving tea quality and taste during the oxidation stage [3], the criticalities in the process are higher with respects to other food production procedures: as a result, the risk of contamination due to harmful pathogens is substantial.

There are no regulations or load limits concerning microbial contamination of teas and infusions, considering that microbiological food safety hazards linked to such food products have rarely been reported [4]. It has to be considered that, although tea contains a natural level of microorganisms, because of its low water activity, the risk related to the growth of microorganisms is not significant, until the product is kept dry: the excessive moisture, indeed, is the main leading to the development of microbiological contamination of tea [5].

In the current research, a hygiene monitoring on 32 different black and green tea confections [*Camellia sinensis* (L.) Kuntze] was performed in order to assess whether the evaluated products, in terms of microbial loads, were in accordance with established guidelines [6, 7]*.*

The research is part of a wider project consisting on a multi-faceted pilot study aimed to analyze 32 brands of European and Italian commercially available tea (16 black teas and 16 green teas) to evaluate: the presence of heavy metals and phthalates (Ferretti V, unpublished data); and adulterations (food fraud) through the a rapid and efficient protocol using DNA barcode (DNA Verity Test) [8].

Our analysis mainly aimed to the microbiological and molecular identification of bacteria and molds in commercial tea samples. In addition to the evaluation of total bacterial count and the research of molds, yeasts and *Escherichia coli*, as suggested by Tea and Herbal Infusions Europe and American Herbal Product Association [6, 7], microbiological analysis was focused on the detection of *Pseudomonas* spp. and *Clostridium perfringens*. Sulphite reducing Clostridia contamination is particularly critical, considering the possibility of the microorganism to produce toxins able to survive to high infusions temperatures [9–11]. Rapid detection methods, through PCR and sequencing protocols, constitute a tailored support to the fast confirmation of food matrices contamination [12]. The molecular characterization of isolated microbial strains consented the definition of the microbial flora in tea and infusions and emphasized the importance of rapid microorganisms identification techniques to prevent foodborne infections.

The study was also focused on the research both on tea dried comminuted leaf matrices present in the teabags (solid sample) and tea infusions (liquid sample) of ochratoxin A (carcinogenic, immunotoxic, nephrotoxic and hepatotoxic mycotoxin), whose migration from tea has been largely reported [13, 14]. The fast detection of ochratoxin A (OTA) produced by molds (especially Aspergilli), through analytical chemistry targeted methodologies (HPLC-FLD), represents a valid assessment of the OTA intake levels via tea: the disposal of such data in fast times is able to support the identification of criticalities before tea products market distribution, reducing the health risk for consumers.

## Methods

### Tea sampling and sample preparation for the laboratory analyses

Tea sampling was the same of De Castro et al. [8]. Briefly, a total of 32 tea packages (*C. sinensis*) were purchased from market in Naples (southern Italy) and online-shops (Table 1). All products are available to consumers also by online-shops and represented by 17 different Italian and international famous brands (7 and 10, respectively). Of these, 16 samples were fermented tea (black tea) and 16 samples were raw tea (green tea). In addition, decaffeinated and soluble samples were also considered. The tea samples were chosen considering both the sales network (supermarkets, drugstores and herbalist’s shops), the price (cheap and expensive), the marketing quality (packaging, publicity and brand) and presence of filters into the packages (except for an accessions of soluble green tea). All information (except for the brand) are reported in Table 1. Tea samples were stored at ambient temperature before the microbiological analyses and mycotoxin characterization. Two different templates were used owing to different analyses: (1) dried for microbiological evaluations (one filter/sample) and mycotoxins (ochratoxin A) detection (5 g/sample); and (2) aqueous infusion for mycotoxins analyses. Briefly, using a microwave, 150 mL of boiled ultrapure water (ROMIL-SpS Super Purity Solvent, ROMIL) was poured into each beaker containing one filter of tea samples and then left to stand (3-8 min) according to manufacturer’s instructions (Table 1). The standards (only water) and extracts were prepared in scrupulously clean glassware. Both microbiological and mycotoxins analysis were performed in duplicate.

**Table 1:** List of the Italian commercialized black and green tea packages analysed in this study (N and V samples, respectively). For each accession is reported information about the marketing quality (high, medium and low), sales network (D = discount supermarket; H = herbalist’s shop; S = supermarket; P = drugstore) and the price (ϵ) [(A), <1ϵ; (B), <1 < ϵ < 2; (C), 2 < ϵ < 4; (D), 4 < ϵ < 6; (E), >6ϵ]. Numbers of filters/package, gr/filter, ingredient and infuse protocol preparation have been copied from the original package

Code	Marketing quality‡	Sales network	Price	n° filters/ package	gr/filter	Label information	Infusions instructions: H_2_O°C, min**
Black teas							
N1	Medium	H	D	2	1.5	Organic black teas leaves of *Camelia sinensis* (L.) Kuntze (100%)	Bo., 5-8
N2	Medium-good	P, H	D	20	2	Tea leaves [*Camellia sinensis* (L.) Kuntze]	Bo., 5-6
N3	Good	S	B	25	1.5	Black tea, aromas	100, 2-3
N4	Low-medium	S	C	15	2	Not reported	Bo., 3
N5	Good	P, H	C	20	1.75	Black certified tea 100%	80, 1-3
N6	Good	S	B	25	1.5	Tea	100, 3-5
N7	Good	S	C	25	1.5	Decaffeinated tea	100, 3-5
N8	Good	S	B	25	1.5	Not reported	Bo., 2-3
N9	Good	S	C	25	2	Black tea (94%), lemon aroma (6%)	100, 3-4
N10	Low	S	B	25	1.75*	Black tea (100%)	Bo., 3-5
N11	Good	S	D	23	1.5	Decaffeinated black tea, caffeine <0.1%	100, 3
N12	Low	S	B	25	1.7	Black tea	Bo., 3
N13	Low	S	B	25	2*	Black tea (95%), natural lemon aroma (5%)	90-100, 3
N14	Low	S	B	25	1.75*	Black tea leaves	Bo., 3-5
N15	Medium	H	C	20	2	Decaffeinated organic black tea, caffeine <0.1%	100, 3-5
N16	Good	S	C	14	5†	Sugar, acidifier (citric acid), decaffeinated tea extract, aromas, lemon juice powdered (0.5%). Gluten free	N.d.
Green teas							
V1	Good	P, H	E	20	2	Pure leaves of green tea (*Camelia sinensis* Kuntze)	Bo., 5-7
V2	Medium	P, H	E	20	2	Green tea leaves (*Camelia sinensis*) (99%), bergamot essential oil (1%)	Bo., 5
V3	Medium	H	D	20	1.5	Organic green tea leave [*Camellia sinensis* (L.) Kuntze] (100%)	Bo., 5-8
V4	Medium	P, H	D	20	1.8*	Green tea leave	Bo., 3-5
V5	Medium-good	P, H	D	20	2	Unfermented organic tea (*Camellia sinensis* L.) leave	Bo., 5-6
V6	Medium	H	C	20	1.75	Green tea certified by Fairtrade	80, 3
V7	Medium	H	D	20	2*	Not reported	Bo., 3-5
V8	Good	S	C	25	1.3*	Green tea (100%)	90, 2-3
V9	Good	S	C	10	1.3*	Green tea, aromas, peel of citruses (2.1%: grapefruit, lemon, lime, orange)	90, 2-3
V10	Good	S	C	10	1.3*	Green tea, aromas, spices 2% (anise, cinnamon, liquorice)	90, 2-3
V11	Good	S	C	10	1.3*	Green tea, aromas, mint (7.9%)	90, 2-3
V12	Good	S	C	25	1.6	Green tea	75, 3
V13	Low	S	B	25	1.75*	Green tea	Bo., 3-5
V14	Low	S	B	25	2*	Organic green tea	80, 3
V15	Low	D	A	20	1.75	Green tea	Bo., 3-5
V16	Medium	S	A	–	125††	Sugar, acidifier (citric acid), green tea soluble extract, aromas, ginseng soluble extract	N.d.

‡, value determined by a questionnaire conducted on 25 people (13 females and 12 males); *, value deducted; **, between a range of two values, the average has been used; †, soluble; ††, soluble power for 1.5 L of infusion; Bo., boiling water, temperature used of 90 °C; N.d., no datum

### Microbiological characterization

#### Sample preparation

The content of each infusion bag tea is singularly weighed into a 50 mL tube and a 1:10 dilution is prepared with 0.9% sterile NaCl solution. Tubes are shaken vigorously for 60 s and vortexed for 10-20 s. The solutions, basing on each microbial research protocol, are plated on solid agar media through spread or pour plating. Sample is plated in two replicates, setting up two ten-fold dilutions per sample. Plates are then incubated at different temperatures and specific times, based on the microorganism or group of microorganisms researched as reported below. A successive DNA barcoding analyses was employed to characterization of microorganisms isolated from selective and non-selective solid enrichment growth media.

#### Total bacterial count isolation

Total bacterial count (TBC) analysis, according to International Organization for Standardization (ISO) procedure [15], consists on pouring 1 mL from the sample in Plate Count Agar (PCA) (Oxoid, Thermo Fisher Scientific Inc., Waltham, MA, USA); PCA plates are incubated at 30 ± 1 °C for 72 h. The totality of colonies is counted. Different morphology colonies are sub-cultured in fresh PCA agar and submitted for molecular analysis.

#### Molds and yeasts isolation

Molds and yeasts research, according to ISO procedure [16], was performed by pour plating 1 mL sample in Rose-Bengal chloramphenicol agar (DRBC, Oxoid, Thermo Fisher Scientific Inc., Waltham, MA, USA); Rose-Bengal agar plates are incubated at 22 ± 1 °C for 72 h. The totality of colonies is counted. Different morphology colonies are sub-cultured in fresh Rose-Bengal agar and submitted for molecular analysis.

#### Escherichia Coli isolation

The presence of *Escherichia coli* was evaluated according to ISO procedure [17]. After sample preparation, 1 mL sample is plated in Tryptone Bile Agar with X-Glucuronide (TBX, Oxoid, Thermo Fisher Scientific Inc., Waltham, MA, USA), using the pour plate method. Plates are incubated at 44 ± 1 °C for 24 h. TBX is a chromogenic medium, able to evidence the presence of *Escherichia coli*, through the growth of typical light-blue colonies.

#### *Pseudomonas* spp. analysis

According to ISO procedure [18], an aliquot of 100 μL sample is spread plated on Pseudomonas Agar Base (Oxoid, Thermo Fisher Scientific Inc., Waltham, MA, USA), then incubated at 37 ± 1 °C for 24 h. The presence of typical colonies on Pseudomonas Agar Base, straw colored with green or brown pigmentation, represents presumptive evidence of *Pseudomonas* spp., further confirmed with additional tests.

#### Clostridium Perfringens research

Sulphite reducing Clostridia and *Clostridium perfringens* research, according to ISO procedure [19], consists on pouring 1 mL from the sample in Perfringens Tryptose Sulphite Cycloserine agar (TSC, Oxoid, Thermo Fisher Scientific Inc., Waltham, MA, USA); TSC plates are incubated at 37 ± 1 °C for 24-48 h. Characteristic colonies show a black precipitate, caused by the reduction of sulfite to sulfide; colonies are often surrounded by a yellow halo. The totality of black colonies is counted. Suspect colonies are submitted for biochemical serotyping analysis which permit to reveal the identity of the presumptive *Clostridium* spp. through the use of RapID ANA II System (Remel, Thermo Fisher Scientific Inc., Waltham, MA, USA). To each available strip, bacterial suspensions prepared from Tryptone Soya Agar (Oxoid, Thermo Fisher Scientific Inc., Waltham, MA, USA) and inoculation fluid (2 mL vials) were inoculated and then incubated at 37 ± 1 °C for 4 h in anaerobic conditions. Eight wells are bifunctional: color changes are interpreted either before and after the addition of provided reagents. The color changes produced by metabolic reactions were interpreted employing ERIC v1.0.771 software (Remel, Thermo Fisher Scientific Inc., Waltham, MA, USA).

#### DNA barcoding analysis

Each sample was analysed by suspending a colony from agar plates into 25 μl of sterile distilled water. The amplification of genomic DNA was performed using a prototype-demo version of a dedicated PCR kit created for Molds, Yeasts and Bacteria (Lyses & PCR-GO KIT - DNA free) (De Castro O., Dept. Biology, Naples, Italy). Starting directly from the bacterial or fungal colony, a rapid extraction is performed employing the kit and DNA purification is following required before proceeding with PCR. The disposal of free-DNA reagents (Hot Start Taq Polymerase and reaction buffer and lysis buffer) results advantageous towards PCR inhibitors that could make difficult the amplification of the templates. The amplification of bacteria was performed using the 63f forward (5′- CAG GCC TAA CAC ATG CAA GTC-3′) and the 1387r reverse (5′-GGG CGG WGT GTA CAA GGC-3′) oligos (Macrogen Inc., Seoul, Rep. of Korea), able to amplify the 16S bacterial rRNA [20]. For the characterization of yeasts and molds, the nuclear internal transcribed spacers (ITS1-2) and 5.8S rDNA were amplified, using ITS5 forward (5′-GGA AGT AAA AGT CGT AAC AAG G-3′) and ITS4 reverse (5′-TCC TCC GCT TAT TGA TAT GC-3′) primers (Macrogen Inc., Seoul, Rep. of Korea) [21]. Amplified samples were purified using PEG8000 precipitation (PEG 20%, NaCl 2.5 M): sequencing reactions were run – using the Di Maio and De Castro method [22] - loading 10 ng/100 bp from each of the purified templates, employing Bright Dye Terminator Cycle Sequencing Kit, based on the use of a fluorescent dye (ICloning, McLab, San Francisco, CA, USA). Reactions were sequenced with a 3130 Genetic Analyzer (Applied Biosystems, Life Technologies, Thermo Fisher Scientific Inc., Waltham, MA, USA), then interpreted with AB DNA Sequencing Analysis version 5.2 software (Applied Biosystems, Thermo Fisher Scientific Inc., Waltham, MA, USA), supported by an additional editing tool, Chromas lite version 2.1.1 (http://technelysium.com.au/?page_id=13). The characterization of the isolated strains was made employing BLASTN ver. 2.2.29 [23], identifying the microorganisms by choosing the highest percentage of identity with a 95% cut-off and a minimum E-value lower than E^−4^.

### Mycotoxin characterization

#### Ochratoxine a determination

Five grams each sample were extracted with 50 mL of CH_3_OH/NaHCO_3_ in water 1%, (70/30) with a 30 min agitation in an orbital shaker at 20 °C. Sample extract was filtered on Whatman n.40 paper (Exacta Optech, San Prospero, MO, Italy). Five mL of the extract were diluted with 20 mL of Phosphate Buffer Solution (PBS), which contains 8 g NaCl, 1.2 g NaHCO_3_, 0.2 g KH_2_PO_4_, 0.2 g KCl in 1 L of water; final pH was adjusted to 7.0 using HCl. The diluted extract was passed through an immunoaffinity column (Vicam, Milford, MA, USA) to lead ochratoxin A (OTA) contained in the extract to the antibody. The elution velocity was set to 2 drop/s. The column was subsequently washed with 10 mL PBS twice and then with 10 mL water. Ochratoxin A was recovered by means 1 mL of methanol that, passing thought the column, denaturated the antibody and solubilized them.

Ochratoxin A determination was made with an HPLC Prominence (Shimadzu, Kyoto, Japan) provided of two pumps LC20AD XR, a degaser DGU20A3, an autosampler SIL20AD XR, an oven CTO10AS VP and a fluormetric detector FP2020 Plus (Jasco, Tokyo, Japan). Separation step was achieved with a Kintex C18 column (Phenomenex, Torrance, CA, USA), 25 cm length, 4.6 mm internal diameter. Samples were injected with a 50 μL volume. For the identification and quantification of ochratoxin A, the mobile phase consisted of methanol/acetonitrile/acetic acid (99/99/2, *v*/v/v) setting a flow rate of 1 mL/min. For detecting the fluorescence, samples were excited at a 333 nm wavelength and by emitting a 466 nm wavelength. The evalutation of the formation of methyl ester with borontrifluoride (BF_3_) resulted functional to confirm the presence of OTA in the samples under analysis: the protocol consisted on drying 1 mL purified extract (which underwent to ochratoxin detection via HPLC) or 1 mL standard solution (with a 4 ng/mL concentration), employing a nitrogen stream; samples were then re-dissolved in 1 mL BF_3_. Following an incubation at 60 °C for 15 min, samples underwent a further HPLC chromatographic analysis. The methylester OTA retention time of was shifted from 10.2 min to 16.8 min instead. The ochratoxin A concentrations were quantified using the calibration curve method, using 2′,4′-difluoro-4-hydroxy-3-biphenylcarboxylic acid (Diflunisal) as internal standard. The samples were analyzed in a PC generated casual sequence. A “blank” sample and a standard were analyzed every 10 sample. Statistical analysis was performed using Statistica software (StatSoft, Oklahoma, USA) and Minitab (Minitab Inc., Pennsylvania, USA). Standard and samples were analysed in triplicate. The normal distribution of data was verified using the Kolmogorov-Smirov test. The comparison between groups (green compared to black teas) was made by means t-test.

## Results

### Microbiological characterization

By applying the guidelines recommended by Tea and Herbal Infusions Europe and American Herbal Product Association, [6, 7], all the batches of teas analyzed were of satisfactory microbiological quality, excepting V5 sample, due to the presence of *Clostridium perfringens* (ranging from 1 to 1 × 10^2^ CFU/g).

Values of total bacterial counts, molds and yeasts research for the samples of black (N_n_) and green (V_n_) tea are shown in Table 2. Green teas total bacterial count presented higher microbial loads compared to black teas. The analysis of black teas brought out that only samples N4, N6 and N9 showed microbial loads higher than 10^3^. Results regarding molds and yeasts never reached the 10^5^ indicated load limit. (Table 2).

**Table 2:** Values of total microbiological counts in the black and green tea samples. The analyses were performed in duplicate (R1 and R2)

	Bacteria				Molds and Yeasts					Bacteria				Molds and Yeasts			
Black tea sample	R1 CFU/g	R2 CFU/g	Mean ± S.D. CFU/g		R1 CFU/g	R2 CFU/g	Mean ± S.D. CFU/g		Green tea sample	R1 CFU/g	R2 CFU/g	Mean ± S.D. CFU/g		R1 CFU/g	R2 CFU/g	Mean ± S.D. CFU/g	
N1	180	200	190	± 14.1	0	0	0	± 0	V1	471	489	480	± 12.7	272	298	285	± 18.4
N2	0	0	0	± 0	0	0	0	± 0	V2	2304	2296	2300	± 5.7	895	905	900	± 7.1
N3	127	113	120	± 9.9	57	43	50	± 9.9	V3	354	346	350	± 5.7	224	236	230	± 8.5
N4	6730	6750	6740	± 14.1	192	208	200	± 11.3	V4	53	47	50	± 4.2	8	12	10	± 2.8
N5	380	400	390	± 14.1	247	233	240	± 9.9	V5	124	118	121	± 4.2	25	35	30	± 7.1
N6	4890	4870	4880	± 14.1	3009	3061	3035	± 36.8	V6	0	0	0	± 0	0	0	0	± 0
N7	0	0	0	± 0	0	0	0	± 0	V7	0	0	0	± 0	0	0	0	± 0
N8	248	232	240	± 11.3	216	204	210	± 8.5	V8	487	493	490	± 4.2	378	402	390	± 17.0
N9	1150	1110	1130	± 28.3	384	396	390	± 8.5	V9	1212	1218	1215	± 4.2	235	245	240	± 7.1
N10	624	696	660	± 50.9	166	154	160	± 8.5	V10	534	546	540	± 8.5	223	237	230	± 9.9
N11	198	202	200	± 2.8	0	0	0	± 0	V11	1437	1423	1430	± 9.9	284	296	290	± 8.5
N12	410	400	405	± 7.1	180	200	190	± 14.1	V12	2626	2634	2630	± 5.7	172	168	170	± 2.8
N13	117	103	110	± 9.9	0	0	0	± 0	V13	17450	17498	17474	± 33.9	4420	4490	4455	± 49.5
N14	685	695	690	± 7.1	434	416	425	± 12.7	V14	623	637	630	± 9.9	254	246	250	± 5.7
N15	189	201	195	± 8.5	0	0	0	± 0	V15	13790	13810	13800	± 14.1	2910	2890	2900	± 14.1
N16	0	0	0	± 0	0	0	0	± 0	V16	0	0	0	± 0	0	0	0	± 0

**Fig. 1:**
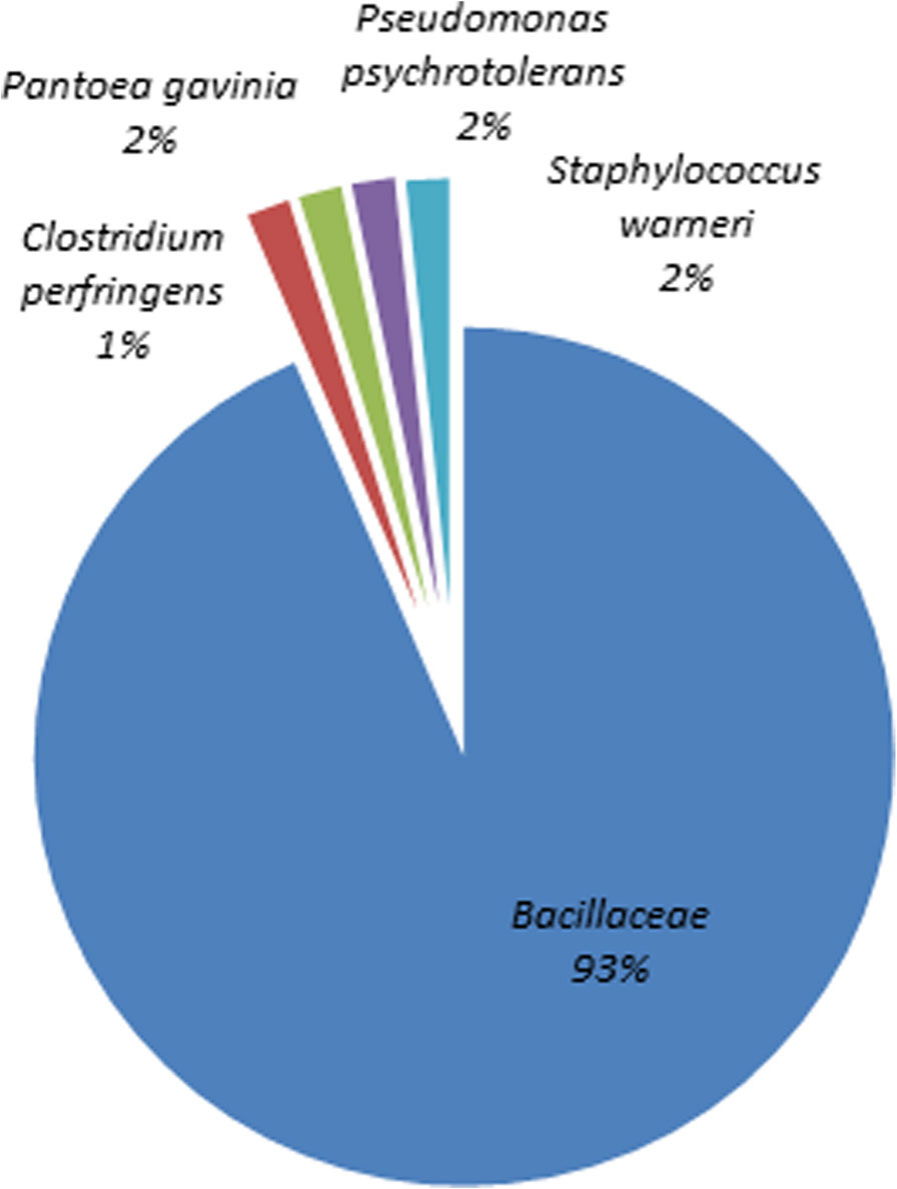
Frequency of the bacterial species isolated from tea samples according to the DNA barcoding characterization

**Fig. 2:**
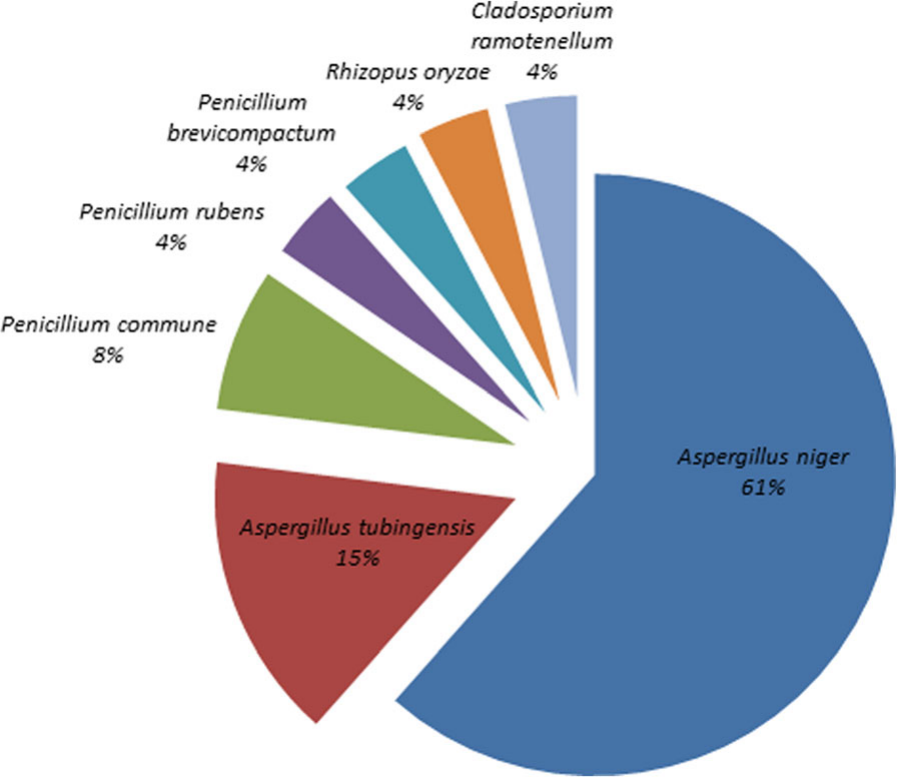
Frequency of the fungal species isolated from tea samples according to the DNA barcoding characterization

**Fig. 3:**
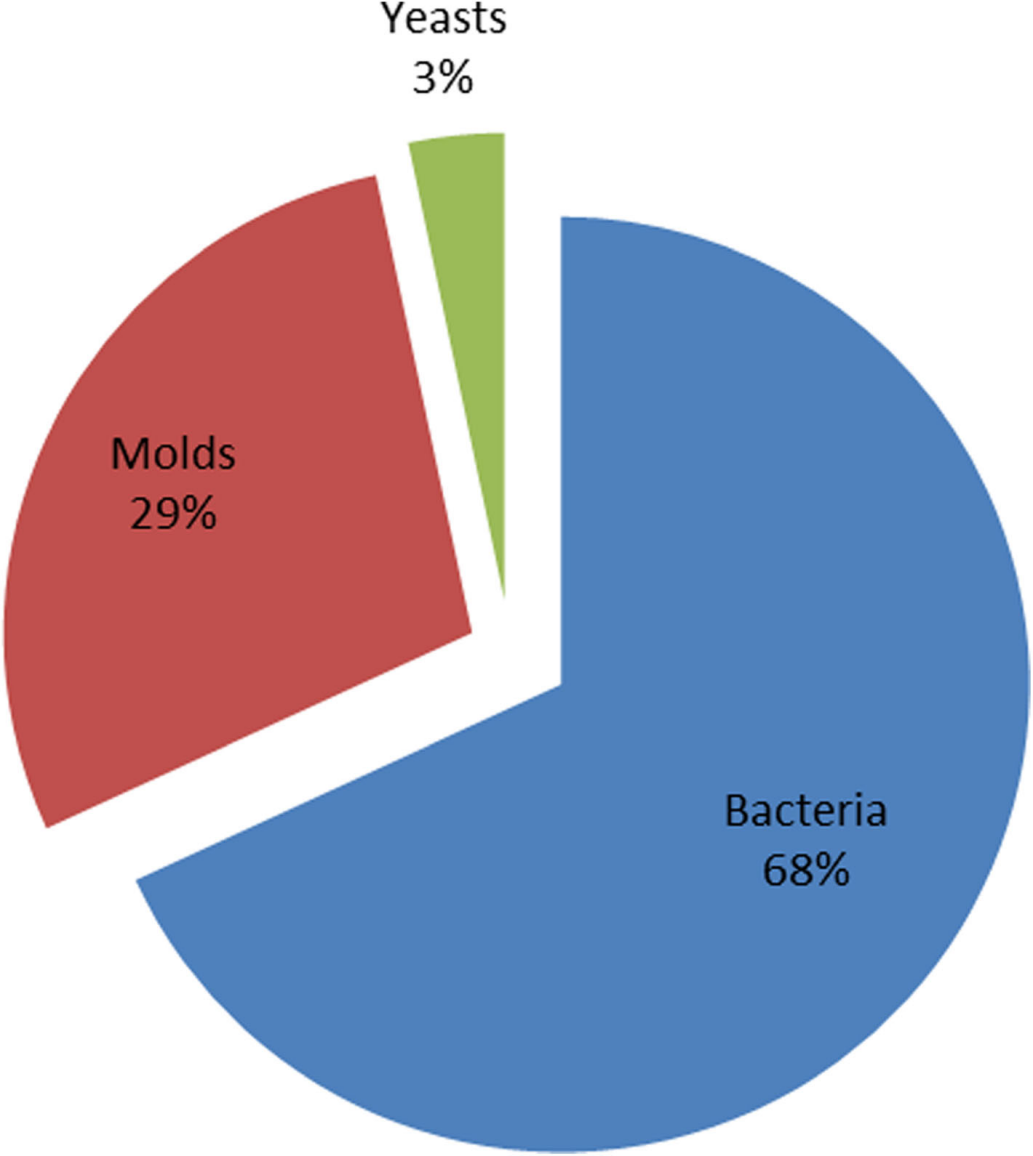
Relative frequency of Bacteria, Molds, and Yeasts isolated from tea samples

According to the DNA barcoding characterization, 91 taxa among bacteria (62 of which 21 species), molds (26 of which 7 species) and yeasts (3 species) were identified, as shown in Table 3, Figs. 1, 2 and 3; 40 taxa were from black and 51 from green teas samples. For both tea varieties, around 70% isolates were bacteria (70% black vs 67% green teas) and the 30% were molds (25% black vs 31% green teas) and yeasts (5% black vs 2% green teas).

**Table 3:** Microbial taxa isolated from black and green tea samples according to the DNA barcoding characterization

Bacteria	Black teas	Green teas	Total	Molds	Black teas	Green teas	Total
*Bacillus amyloliquefaciens*	3	4	7	*Aspergillus niger*	6	10	16
*Bacillus aryabattai*	0	2	2	*Aspergillus tubingensis*	1	3	4
*Bacillus cereus*	4	5	9	*Penicillium commune*	1	1	2
*Bacillus circulans*	1	0	1	*Penicillium rubens*	0	1	1
*Bacillus drentensis*	0	1	1	*Penicillium brevicompactum*	0	1	1
*Bacillus endophyticus*	0	2	2	*Rhizopus oryzae*	1	0	1
*Bacillus licheniformis*	3	3	6	*Cladosporium ramotenellum*	1	0	1
*Bacillus megaterium*	0	2	2	Total (7 species)	10	16	26
*Bacillus methylotrophicus*	0	1	1				
*Bacillus pseudomycoides*	1	0	1				
*Bacillus pumilus*	2	2	4				
*Bacillus subtilis*	3	6	9				
*Bacillus tequilensis*	0	2	2	Yeasts	Black teas	Green teas	Total
*Bacillus thuringensis*	7	1	8	*Cryptococcus neoformans*	0	1	1
*Clostridium perfringens*	0	1	1	*Rhodotorula mucillaginosa*	1	0	1
*Paenibacillus cineris*	1	0	1	*Sporidiobolis ruineniae*	1	0	1
*Paenibacillus lactis*	0	1	1	Total (3 species)	2	1	3
*Paenibacillus taichungens*	0	1	1				
*Pantoea gaviniae*	1	0	1				
*Pseudomonas psychrotolerans*	1	0	1				
*Staphylococcus warneri*	1	0	1				
Total (21 species)	28	34	62

The identified bacterial species are listed in Table 3. Most bacteria (68% total isolates) belong to the *Bacillaceae* family (93%, 58 taxa) (Fig. 1 and Table 3), with a prevalence of *Bacillus thuringensis* (seven isolates) and *Bacillus cereus* (four isolates) in black teas, and a prevalence of *Bacillus subtilis* (six isolates), *Bacillus cereus* (five isolates) and *Bacillus amyloliquefaciens* (four isolates) in green teas. Considering black tea samples, beyond Bacilli and Paenibacilli, three taxa from different families were identified: *Pantoea gavinia*, *Pseudomonas psychrotolerans* and *Staphylococcus warneri*. According to the bacterial families, green teas evidenced a bacterial diversity, with respects to *Bacillaceae*, only in one case (V5 sample) where the potentially pathogenic spore producing *Clostridium perfringens* was isolated.

Data regarding molds (Figs. 2 and 3 and Table 3) evidence that the majority of isolates belongs to *Aspergillus* genus, mostly *Aspergillus niger* and *Aspergillus tubingensis*. While, from black teas, taxa of *Cladosporium ramotenellum* (N8 sample) and *Rhizopus oryzae* (N10 sample) were identified, mostly *Penicillium* species (*Penicillium commune,* V4 sample; *Penicillium rubens,* V5 sample*;* and *Penicillium brevicompactum,* V15 sample) were isolated from green tea samples. Only three yeasts were identified: *Cryptococcus neoformans* from green tea sample (V15 sample); *Rhodotorula mucillaginosa* and *Sporidiobolis ruineniae* isolated from black tea (N6 sample) (Table 3).

### Mycotoxin characterization

#### Ochratoxine a: Infusion evaluation

OTA was detectable in 68.8% of the green tea infuses and in 37.5% of the black tea infuses. The mean concentration was higher in the black tea samples, even not significantly (0.06 ± 0.03 Vs 0.04 ± 0.02 [μg/L] of green tea). The OTA transfer ratio during the infusion process ranged between 33.65 ± 4.37% for the black tea and 54.47 ± 14.48% for the green tea (Table 4).

**Table 4:** Ochratoxin A concentration in the tea infusions samples

Sample (Black tea)	Ochratoxin A (ng/L)	Sample (Green tea)	Ochratoxin A (ng/L)
N01	0.1 ± 0.0	V01	0.2 ± 0.0
N02	0.1 ± 0.0	V02	0.1 ± 0.2
N03	0.8 ± 0.2	V03	0.6 ± 0.1
N04	0.4 ± 0.1	V04	0.8 ± 0.2
N05	0.4 ± 0.1	V05	0.4 ± 0.1
N06	0.1 ± 0.0	V06	0.4 ± 0.1
N07	0.1 ± 0.0	V07	0.1 ± 0.2
N08	0.1 ± 0.0	V08	0.6 ± 0.1
N09	0.1 ± 0.0	V09	0.6 ± 0.1
N10	0.1 ± 0.0	V10	0.4 ± 0.1
N11	0.1 ± 0.0	V11	0.1 ± 0.0
N12	0.6 ± 0.1	V12	0.4 ± 0.1
N13	1.0 ± 0.2	V13	0.2 ± 0.0
N14	0.4 ± 0.1	V14	0.2 ± 0.0
N15	0.1 ± 0.0	V15	0.1 ± 0.0
N16	0.1 ± 0.0	V16	0.1 ± 0.0

#### Ochratoxine a: Leaf evaluation

**Fig. 4:**
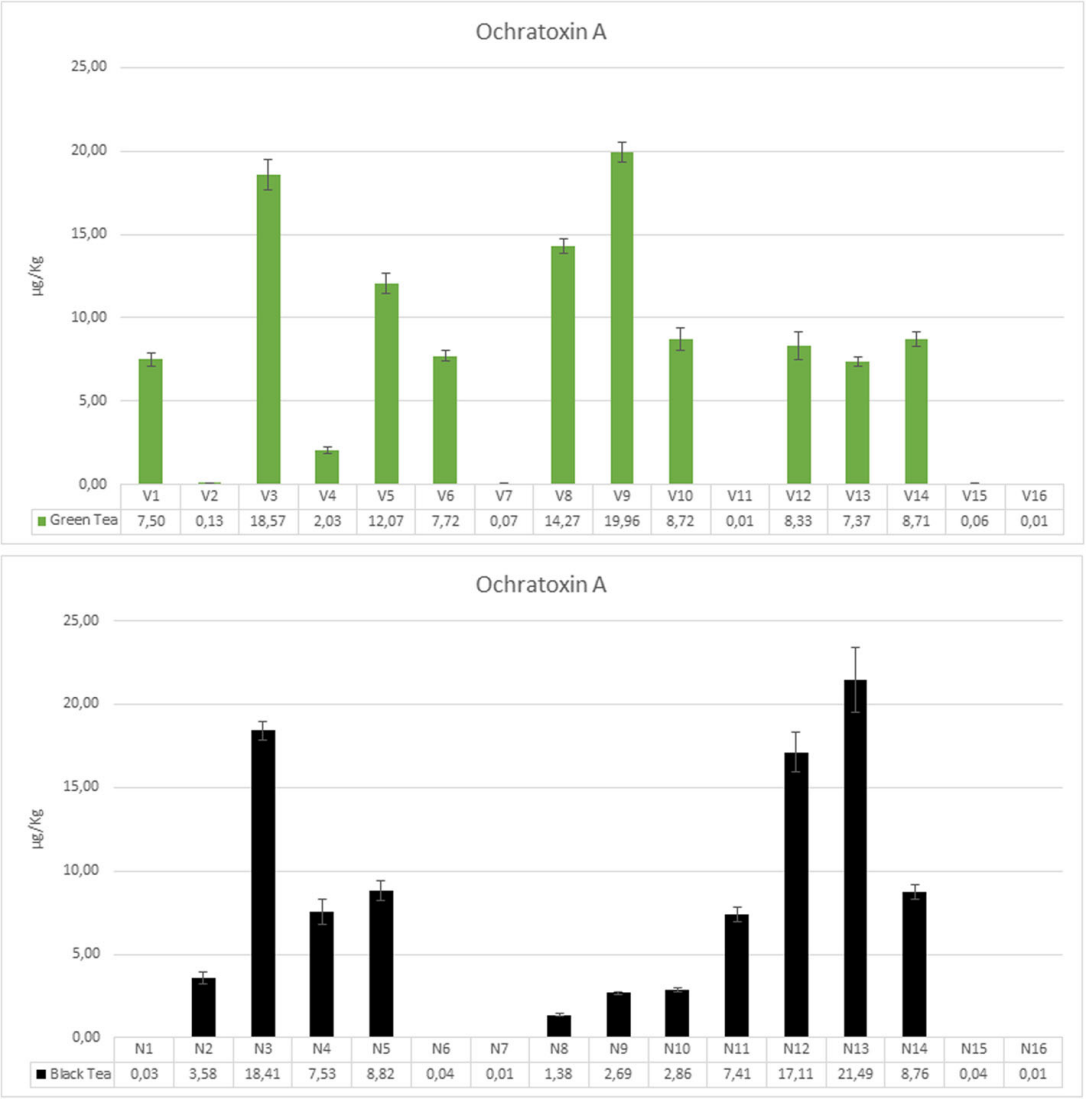
Ochratoxin A concentrations values (μg/kg) in the dried comminuted leaf matrices of the black and green teabags. Further details in Table 5

Ochratoxine A (OTA) was detectable in 82.5% of green and black tea samples (Fig. 4 and Table 5). OTA mean concentration was lower in black tea compared with green tea, although not statistically significant (6.26 ± 7.13 vs 7.22 ± 6.6 [μg/Kg]). The highest concentration was found in a black tea sample (21.49 μg/Kg, N13 sample) (Fig. 4 and Table 5). All the instant soluble samples had not detectable levels of OTA (< 0.01 μg/Kg).

**Table 5:** Ochratoxin A concentration in the dried leaves matrices of teabags

Sample (Green tea)	Ochratoxin A ± S.D. (μg/Kg)	Sample (Black tea)	Ochratoxin A ± S.D. (μg/Kg)
V1	7.5 ± 0.37	N1	0.03 ± 0.00
V2	0.13 ± 0.01	N2	3.58 ± 0.36
V3	18.57 ± 0.93	N3	18.41 ± 0.55
V4	2.03 ± 0.18	N4	7.53 ± 0.75
V5	12.07 ± 0.60	N5	8.82 ± 0.62
V6	7.72 ± 0.31	N6	0.04 ± 0.00
V7	0.07 ± 0.00	N7	0.01 ± 0.00
V8	14.27 ± 0.43	N8	1.38 ± 0.10
V9	19.96 ± 0.60	N9	2.69 ± 0.08
V10	8.72 ± 0.70	N10	2.86 ± 0.14
V11	0.01 ± 0.00	N11	7.41 ± 0.44
V12	8.33 ± 0.83	N12	17.11 ± 1.20
V13	7.37 ± 0.29	N13	21.49 ± 1.93
V14	8.71 ± 0.44	N14	8.76 ± 0.44
V15	0.06 ± 0.00	N15	0.04 ± 0.00
V16	0.01 ± 0.00	N16	0.01 ± 0.00

## Discussion

### Microbiology

According to our data, green teas evidenced higher microbial loads (1.7 × 10^4^ CFU/g) with respect to black teas. Similarly, isolated molds and yeasts, although widely within the 10^5^ CFU/g recommended limit [6, 7], highlight a numerousness, which, for some samples, could justify possible alterations of the product.

Biological tea products analyzed (ten samples: N1, N2, N5, N13, N15, V3, V5, V6, V7, V14), compared to our non-bio tea matrices (22 samples), present lower microbial loads, though variable and, in 30% cases, the product was microbiologically sterile. Teas not reporting the biological certification, presented a lower microbiological sterility rate (14%). It would be useful to verify the processes to which such products are submitted before commercialization, given that, without a thermal or likewise treatment, it is not easily achievable. The generic 2:1 bacteria:fungi ratio, obtained through molecular biology evaluations, does not result peculiar, considering that the majority of food products of plant origin show such rates [24]. On the other hand, bacterial and fungal species differentiation resulted important: especially for molds, the proper classification allows the identification of aflatoxins producing species, able to resist to teas and infusions extraction process.

According to our molecular analyses, the highest proportion of isolated strains from green tea (51 strains), compared to black tea samples (40 strains), confirmed the higher microbial loads registered for this variety of tea. Moreover, the 70:30 bacteria:molds/yeasts ratio traces the 2:1 bacteria:fungi ratio from the microbiological analysis. The available data concerning identified bacterial strains confirm previous researches reporting *Bacillaceae* and *Paenibacillaceae* together with *Enterobacteriaceae*, as the most frequently identified bacterial species [25]. *Bacillaceae* are easily isolated from food matrices and generally do not constitute a risk for the health of consumers. Over the Bacilli and Paenibacilli, the bacterial diversity was represented by the isolation of: *Pseudomonas psychrotolerans*, *Staphylococcus warneri*, *Pantoea gaviniae*. The isolation of *Pseudomonadaceae* and *Staphylococcaceae* can be considered common, since these bacteria are naturally present in soil [26] and, however, such microorganisms could not survive at high temperatures [27, 28]. On the other hand, interesting resulted the isolation of the facultatively anaerobic strain of *Pantoea gaviniae,* from sample N9: a recent study demonstrated that *Pantoea gaviniae*, together with *Pantoea calida*, are the closest phylogenetic relatives of *Pantoea theicola*, which is typical from black tea: this strain was isolated from black tea extract heated at 90 °C [29], indicating the potential of the isolated microorganism to resist not only to anaerobic conditions, but also to high temperatures [30]. *Pantoea* sp. is an opportunistic pathogen, isolated from different hosts, belonging to the Enterobacteriaceae family, usually employed as indicators for potential contaminations and not proper storage of food products [30]. One *Clostridium perfringens* strain, whose identification was also confirmed through molecular biology analysis, was isolated from sample V5, biological green tea, with a 50 CFU/g bacterial load. *Clostridium perfringens* is an anaerobic obligate bacterium, surviving to low moisture and oxygen levels, well known for its ability of producing toxins, which result crucial for the onset of foodborne pathologies [31]. Since teas are dehydrated, it can be hypothesized that the isolated germs were spores forms, thus not metabolically active for toxins production. In this case, the storage of the product in humidity-free environments is critical as reported by [9–11].

Fungi were isolated from the majority of samples: four fungal genera (*Aspergillus*, *Penicillium*, *Rhyzopus* and *Cladosporium*) and seven species were detected. The most prevalent microrganisms were *Aspergillus niger* and *A. tubingensis*, followed by *Penicillium* (*Penicillium commune*, *Penicillium brevicompactum* and *Penicillium rubens*), *Cladosporium* and *Rhyzopus* species. Studies regarding the research of molds in teas and herbal infusions reported that *Aspergiullus* and *Penicillium* species generally prevail, followed by microrganisms belonging to *Cladosporium*, *Alternaria*, *Rhizopus*, *Absidia* and *Trichoderma* genera [32].

*Aspergillus niger*, together with *Aspergillus tubingensis*, belonging to the black Aspergilli group, represent the most frequently isolated fungal contaminants in our tea samples. The human health risk linked to these fungal species lays in their ability to produce the mycotoxins ochratoxin A (OTA), nephrotoxic and carcinogenic toxins which some microorganisms from *Aspergillus* and *Penicillium* genera are able to produce [33]. Beyond *Aspergillus* genus, *Penicillium* species (especially *Penicillium commune*) are commonly isolated from various food samples, included tea [34, 35]. *Penicillium brevicompactum* is an indoor mould producing a mycotoxin, the mycophenolic acid (MPA). This fungal specie can be isolated from several food matrices [36]. *Rhizopus oryzae* is a fungal specie which constitutes saprotroph in soil, dung, and spoiled vegetation and it generally used for the fermentation of specific foods [37]; in addition, several species are opportunistic and are able to infect humans [38]. *Cladosporium ramotenellum* is a widely distributed saprobic mold and has been recently isolated from hypersaline water [39], but also from food matrices (cheese) [40]. In general, *Rhizopus* and *Cladosporium* genera, as much as *Penicillium*, being soil-dwelling microorganisms, are not considered risky for consumers, especially considering the tea as a food matrix.

Yeasts resulted the least isolated microorganisms from the two varieties of tea: two strains were isolated from black tea samples, *Rhodotorula mucilaginosa* and *Sporidiobolus ruineniae* (both isolated from sample N6), one from green tea, *Cryptococcus neoformans*. *Rhodotorula mucilaginosa* is a basidiomycetous yeast, which can be isolated from air and soil, but also from human skin, stool and food. Even though the majority of *Rhodotorula* species are non-pathogenic, *Rhodotorula mucilaginosa* in particular, results the most frequently isolated from human infections [41]. *Sporidiobolus ruineniae* has been previously isolated from fermented tea [42], but possesses a low resistance to high temperatures (optimum growth range from 15 °C to 35 °C) [42] such as those employed for tea and infusions heating. The analysis of V15 green tea evidenced the presence of *Cryptococcus neoformans*, an encapsulated yeast, opportunistic fungal pathogen, which is able to live in animal and plants. It is a spore-producing microorganism, causing pulmonary mycosis, and it is often found in bird excrements. Some researches were conducted in demonstrate the antifungal activity of tea extracts towards clinical strains of *Cryptococcus neoformans* [43, 44]: for this reason, it can be hypothesized that, being commonly found in soil samples, this yeast represents a risky microorganism, especially for immunocompromised individuals.

By comparing the results with the available survey from EHIA (European Herbal Infusions association) [45], it can be evidenced that aerobic plate count (total bacterial count) results in the majority of cases between the 10^6^-10^8^ UFC/g range, compared to our samples, whose microbial loads does are not higher than 10^5^ UFC/g; molds and yeasts counts result lower (≤10^3^ UFC/g) than the available data from EHIA (10^3^-10^6^ UFC/g range) [45]. An available research concerning tea microbiome supports the present study outcomes, reporting the majority of isolates belonging to *Bacillus* genus for Bacteria, and to *Aspergillus* and *Cladosporium* genera for molds [46].

### Mycotoxins

Various species of molds are isolated from analyzed tea samples. *Aspergillus niger*, whose 16 isolates have been characterized in this study, has been associated with tea [47]. It was moreover reported that 7% of *Aspergillus niger* isolated from herbal teas produce OTA [33]. In Europe there are not regulations for the OTA concentration in tea. Only coffee is regulated by Commission Regulation (EC) No 1881/2006 with a maximum allowed concentration of 5 μg/Kg in roasted coffee and ten in green coffee. With respect to these limits, our positive samples seems to be substantially contaminated. Other studies report high OTA concentration in tea. Haas et al. [34] report a 94.7 μg/Kg concentration of OTA in a Pu-erh tea sample, while Malir et al. [13] report a concentration of 250 μg/Kg in a black tea sample and a mean OTA concentration of 33.1 μg/Kg based on 12 samples of black tea. The OTA transfer in beverage after the infusion procedure have demonstrated a high influence from the infuse pH [14].

Based on Commission Regulation (EC) No 1881/2006 indications, available data show that seven black tea samples and ten green tea samples (overall, half the samples evaluated) present ochratoxin A concentrations higher than the 5 μg/Kg limit. Analysing green tea results, six samples reported OTA values between 7 and 9 μg/Kg, four samples over 9 μg/Kg, with V3 and V9 showing concentrations higher than 14 μg/Kg (three times the indicated limit). Black tea results registered, for three samples, OTA values between 7 and 9 μg/Kg and, for samples N3, N12 and N13, OTA concentrations higher than 17 μg/Kg. The observed values were different in the green and black samples; this can be explained by differences in the final pH. Since the infuse was prepared with pure water to minimize the cross contamination and to standardize the procedure, the high difference in the ochratoxin A transfer can suggest to investigate this phenomenon in real conditions using drinking water that can modify the tea soluble acids.

## Conclusion

Hygiene monitoring performed on tea samples brought out that such food product does not contain microbial loads which can be harmful for consumers. On the other hand, the attention has to be focused on mycotoxins migration, especially ochratoxin A, whose concentrations resulted over the indicated limits for food products in the 50% analyzed samples. The proper storage of the product is however crucial during the production chain, but particularly for retailers and consumers: storage in humid environment constitutes the highest human health risk, for tea and tea products, triggering the growth of potentially pathogenic microorganisms, especially those able to produce toxins. The integration of standardized cultural methods with molecular characterization of microorganisms is able to consent the detection of highly pathogenic strains, employing faster and more precise protocols, aiming to the optimization of health risk assessment.

## Abbreviations

BF3: Borontrifluoride
*C. sinensis*: *Camellia sinensis*
DRBC: Rose-Bengal chloramphenicol agar
HPLC-FLD: High-performance liquid chromatography with fluorescence detection
ISO: International Organization for Standardization
ITS: Internal Transcribed Spacer
NaCl: Sodium Chloride
OTA: Ochratoxin A
PCA: Plate Count Agar
PCR: Ploymerase Chain Reaction
PEG: Polyethylene glycol
TBC: Total Bacterial Count
TBX: Tryptone Bile Agar with X-Glucuronide
TSC: Tryptose Sulphite Cycloserine agar
